# Acute Neonatal Suppurative Parotitis Due to Co-Infection by Non-Typable Beta-Hemolytic Streptococci and Escherichia coli: A Case Report

**DOI:** 10.7759/cureus.8279

**Published:** 2020-05-25

**Authors:** Ribal Bou Shaheen, Mabelle Al Khoury, Souad Seif, Huneida Hamzeh, Aouni Alameddine

**Affiliations:** 1 Department of Pediatrics, Haykel Hospital, Tripoli, LBN; 2 Faculty of Medicine, Beirut Arab University, Beirut, LBN

**Keywords:** neonatal suppurative parotitis, salivary gland, non-typable beta-hemolytic streptococcus, escherichia coli, case report

## Abstract

Neonatal suppurative parotitis (NSP) is a very rare disease of the salivary glands, usually involving the parotids. It is mostly due to Staphylococcus aureus bacterial infection among patients with predisposing factors, and management mainly relies on hydration and intravenous antibiotics with an excellent prognosis. Herein, we present a case of an 11-day-old female baby who presented with left periauricular edema and tenderness associated with fever that was later diagnosed as NSP due to both non-typable beta-hemolytic streptococci and Escherichia coli. Antibiotics therapy (vancomycin and gentamicin) was started, and the patient recovered completely after nine days. The CAse REport (CARE) guidelines were followed for reporting our case. Our case reiterates the fact that NSP should be in the differential diagnosis of any neonate presenting with fever and parotid area swelling. Prompt diagnosis is necessary for early management with antibiotics and to avoid complications.

## Introduction

Neonatal suppurative parotitis (NSP) is a rare disease of the salivary glands, usually involving the parotids, with an estimated prevalence of 3.8 per 10,000 cases [1,2]. In four decades, only 32 cases of NSP have been identified, as reported in a study by Spiegal et al [2]. NSP is most commonly caused by Staphylococcus aureus bacteria but may also be due to other bacterial isolates such as Escherichia coli, Pseudomonas aeruginosa, and group B streptococci [3]. Pathologically, the development of NSP has been assumed to occur due to hematogenous seeding of the gland or ascending infection from the oral cavity through the Stensen's duct, which leaves the parotid gland in front of the pinna, and runs beneath the zygomatic arch over the masseteric muscle [4]. Some factors that increase the risk of acquiring the infection include trauma to the oral cavity, decreased immunity or immunodeficiency, dehydration, obstruction of the Stensen's duct, structural glandular abnormalities, low birth weight, sepsis, nasogastric intubation, and malnutrition [3]. Management relies mainly on hydration and intravenous antibiotics and rarely needs surgical intervention.

We report the case of an 11-day-old Lebanese female baby diagnosed with neonatal suppurative parotitis due to non-typable beta-hemolytic streptococcus bacterial infection and managed successfully with antibiotics. In addition, we discuss the differential diagnoses and therapeutic approaches implemented in treating this case and similar cases. This case report was conducted and reported in accordance with the CAse REport (CARE) guidelines for reporting case reports.

## Case presentation

An 11-day-old full-term baby girl born at 40 weeks of gestation by C-section and birth weight of 2780 g to a 33-year-old mother (G1P0A0; who claimed a smooth course of pregnancy (except for tooth extraction due to dental abscess a week prior to delivery treated with amoxicillin-clavulanic acid antibiotic) with no perinatal and postnatal complications) was admitted to the emergency department due to one day of fever (two times reaching above 38°C as measured by the mother at home), and swelling in the left periauricular region. Facial or head trauma, maternal mastitis, or other maternal skin infection (as the patient was exclusively breastfed, and this might be a source of infection) were excluded after the initial assessment. No perinatal complications and no relevant maternal or familial history had been reported.

On physical examination, the patient was active. Local inflammatory signs at the left periauricular region were noticed, including hotness, redness, and tenderness, with rigidity covering an area of approximately 1 × 1 cm. Mouth and ear examinations showed purulent discharge at the Stensen's duct. Swab and blood cultures were taken.

Laboratory tests showed a hemoglobin level of 14.3 g/dL (reference range: 12.5-21.0 g/dL), hematocrit level of 43.0% (reference range: 39.0% to 60.0%), white blood cell count of 25.3 × 103/μL (reference range: 5.0 to 21.0 × 103/μL) with 63.0% neutrophils, 23.1% lymphocytes, 11.5% monocytes, 1% basophils, and 1.4% eosinophils, platelets count of 491 × 103/μL (reference range: 150 to 450 × 103/μL), and C-reactive protein level of 9 mg/L (reference range: 0 to 6 mg/L). Serum alpha-amylase concentration was 11 IU/L (reference range: 25 to 125 IU/L). Other laboratory tests, including electrolytes, urea, and creatinine, were not significant.

**Figure 1:**
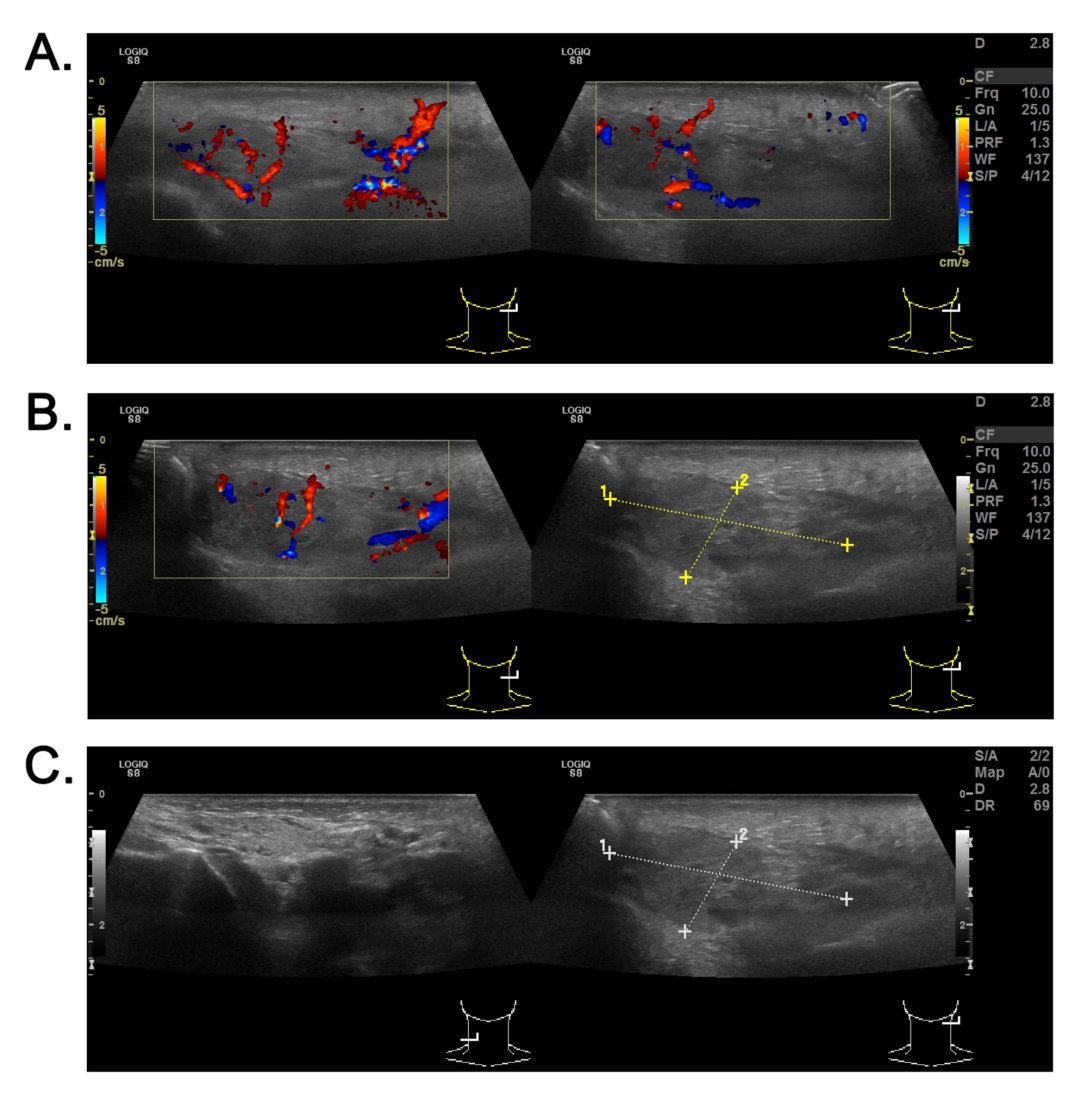
Ultrasound of the left parotid and preauricular region showing enlarged and edematous left parotid gland (A) Left parotid gland with increased internal Doppler activity suggesting left parotitis. (B and C) The left parotid gland measured 3.66 × 1.57 cm

Ultrasound examination of the left parotid and preauricular region revealed an enlarged and edematous left parotid gland with increased internal Doppler activity suggesting left parotitis (Figure 1). The left parotid gland measured 3.66 × 1.57 cm. Adjacent subcutaneous edema was noted. The right parotid gland appeared normal in size and echotexture with normal Doppler activity and no signs of inflammatory changes.

Considering the clinical presentation and ultrasound findings, the girl was diagnosed with left acute suppurative parotitis. She was hospitalized, and an intravenous empirical antibiotic therapy with vancomycin (50 mg/kg/day) and gentamicin (7.5 mg/kg/day) was initiated. Moreover, analgesia with paracetamol and intravenous fluid therapy was implemented. After one day of parenteral therapy, her fever subsided, and on the fourth day of treatment, the parotid swelling significantly diminished. Blood cultures returned negative while the Stensen's duct exudate culture returned positive for non-typable beta-hemolytic streptococci and Escherichia coli sensitive to the instituted antibiotic (Table 1).

**Table 1:** Antibiotic susceptibility profile of the non-typable beta-hemolytic streptococci and Escherichia coli *Antibiotic used in patient treatment. Abbreviations: R, resistant; S, sensitive

Antibiotics	Antibiotic susceptibility	
	Non-typable beta-hemolytic streptococci	Escherichia coli
Ampicillin	S	S
Amoxicillin/clavulanic acid	S	S
Cefalexin	S	S
Cefepime	S	S
Cefixime	S	S
Cefoxitin	S	S
Ceftazidime	S	S
Ceftriaxone	S	S
Cefuroxime	S	S
Piperacillin	S	S
Piperacillin/tazobactam	S	S
Ticarcillin	S	S
Imipenem	S	S
Meropenem	S	S
Ertapenem	S	S
Aztreonam	S	S
Amikacin	S	S
Gentamicin	S*	S*
Ciprofloxacin	S	S
Levofloxacin	S	S
Tigecycline	S	S
Trimethoprim/sulfamethoxazole	S	S
Penicillin	R	S
Vancomycin	S*	S*
Teicoplanin	S	S
Clindamycin	S	S
Erythromycin	R	S
Moxifloxacin	R	S
Tetracycline	R	S

The patient was discharged from the hospital after nine days of treatment with regression of edema and complete recovery. On the follow-up visit two months later, the patient was doing well with no reported sequelae.

## Discussion

Infections of the salivary gland are not common in neonates, but if they occur, they involve the parotid gland in most cases. NSP is a rare disease that affects the parotids unilaterally (in 77% of cases) accompanied by swelling (77%) with or without redness and exuded pus from the ipsilateral Stensen's duct [2,5]. The major causative agent is Staphylococcus aureus (61%) [5]. Diagnosis of NSP is mainly clinical, although ultrasound examination of the parotid and preauricular region is usually performed to confirm the diagnosis and rule out abscess formation [5]. Three diagnostic criteria of suppurative parotitis were described in 1970 by David et al. that include: (i) parotid swelling, (ii) purulent exudation from the Stensen's duct, and (iii) growth of pathogenic bacteria in a culture of the pus [6]. Nevertheless, in any suspected NSP, a general sepsis workup must be done along with ultrasound of the parotid gland and otolaryngology and infectious disease consultations [3].

In a study by Ismail et al. published in 2013, including 44 reported cases of NSP between 1970 and 2013, men constituted 77% of patients [5]. Fever was only seen in 47% of cases, and prematurity was present in a third of the patients. In the same study, it was shown that the mainstay of therapy was a combination of anti-staphylococcal antibiotics with either an aminoglycoside or a third-generation cephalosporin [5]. Since medical therapy renders high success rates, there is no need for surgical intervention in most cases [3].

Unlike most reported cases, our patient was a girl [5,7]. The patient was febrile on admission with swelling in the left parotid region. She had peripheral leukocytosis with increased neutrophil count suggestive of an infectious process due to a bacterial pathogen. The differential diagnosis included lymphadenitis, cellulitis, mumps parotitis, soft-tissue abscess, mastoiditis, and non-infectious etiologies such as congenital anomalies (hemangioma or venolymphatic malformation of the parotid gland) [3].

Although Staphylococcus aureus is the most frequently isolated pathogen in cultures, pus cultures in our case were positive for both non-typable beta-hemolytic streptococci and Escherichia coli [2,5]. Interestingly, and unlike most cases reported in world literature, pus culture was positive in our patient to two bacteria [2,5]. She was breastfed, raising the concern that she might have contracted the infection from her mother (via maternal mastitis or other maternal skin infection); however, the latter was excluded. To make and confirm the diagnosis, ultrasound was performed to exclude other factors that might have predisposed the patient to develop NSP like Stenson's duct abnormality, sialolith, and parotid gland neoplasm. In our case, vancomycin and gentamicin were given for nine days along with aggressive hydration, after which the patient was discharged home with complete recovery after two months follow-up [3]. In some reports, potential complications of NSP have been reported, such as osteomyelitis of the mandible or temporomandibular joint, thrombophlebitis of the jugular vein, respiratory obstruction, and sepsis [3].

## Conclusions

NSP is very rare in neonates. Our case reiterates the fact that NSP should be in the differential diagnosis of any neonate presenting with fever and parotid area swelling. Prompt diagnosis is necessary for early management with antibiotics and to avoid complications.

## References

[1] Sabatino G, Verrotti A, de Martino M, Fusilli P, Pallotta R, Chiarelli F (1999). Neonatal suppurative parotitis: a study of five cases. Eur J Pediatr.

[2] Spiegel R, Miron D, Sakran W, Horovitz Y (2004). Acute neonatal suppurative parotitis: case reports and review. Pediatr Infect Dis.

[3] D’Souza JN, Geary C, Mukerji S (2012). Neonatal parotid gland enlargement: is it suppurative parotitis? A case report. Am J Case Rep.

[4] Bradley PJ (2002). Microbiology and management of sialadenitis. Curr Infect Dis Rep.

[5] Ismail EA, Seoudi TM, Al-Amir M, Al-Esnawy AA (2013). Neonatal suppurative parotitis over the last 4 decades: report of three new cases and review. Pediatr Int.

[6] David RB, O'Connel EJ (1970). Suppurative parotitis in children. Am J Dis Child.

[7] Moradi M (2014). Neonatal suppurative parotitis: a case report. Iran J Pediatr.

